# The first generation of a regional-scale 1-m forest canopy cover dataset using machine learning and google earth engine cloud computing platform: A case study of Arkansas, USA

**DOI:** 10.1016/j.dib.2023.109986

**Published:** 2023-12-30

**Authors:** Hamdi A. Zurqani

**Affiliations:** a University of Arkansas Division of Agriculture, Arkansas Forest Resources Center, University of Arkansas System, 110 University Court, Monticello, AR 71656, USA; b College of Forestry, Agriculture, and Natural Resources, University of Arkansas at Monticello, 110 University Court, Monticello, AR 71656, USA

**Keywords:** Remote sensing, Geospatial analysis, Cloud-based approach, Supervised classification, Random forest classifier, NAIP imagery, Forest cover extraction

## Abstract

Forest canopy cover (FCC) is essential in forest assessment and management, affecting ecosystem services such as carbon sequestration, wildlife habitat, and water regulation. Ongoing advancements in techniques for accurately and efficiently mapping and extracting FCC information require a thorough evaluation of their validity and reliability. The primary objectives of this study are to: (1) create a large-scale forest FCC dataset with a 1-meter spatial resolution, (2) assess the regional spatial distribution of FCC at a regional scale, and (3) investigate differences in FCC areas among the Global Forest Change (Hansen et al., 2013) and U.S. Forest Service Tree Canopy Cover products at various spatial scales in Arkansas (i.e., county and city levels). This study utilized high-resolution aerial imagery and a machine learning algorithm processed and analyzed using the Google Earth Engine cloud computing platform to produce the FCC dataset. The accuracy of this dataset was validated using one-third of the reference locations obtained from the Global Forest Change (Hansen et al., 2013) dataset and the National Agriculture Imagery Program (NAIP) aerial imagery with a 0.6-m spatial resolution. The results showed that the dataset successfully identified FCC at a 1-m resolution in the study area, with overall accuracy ranging between 83.31% and 94.35% per county. Spatial comparison results between the produced FCC dataset and the Hansen et al., 2013 and USFS products indicated a strong positive correlation, with R^2^ values ranging between 0.94 and 0.98 for county and city levels. This dataset provides valuable information for monitoring, forecasting, and managing forest resources in Arkansas and beyond. The methodology followed in this study enhances efficiency, cost-effectiveness, and scalability, as it enables the processing of large-scale datasets with high computational demands in a cloud-based environment. It also demonstrates that machine learning and cloud computing technologies can generate high-resolution forest cover datasets, which might be helpful in other regions of the world.

Specifications Table

**Table:** 

Subject	Forest Resources
Specific subject area	Remote Sensing, GIS, Forest Canopy Cover, Mapping and Evaluation
Type of data	Figures Tables Supplementary data GEE App
How data were acquired	All the data processing was conducted using the cloud-computing technology in the GEE platform (https://earthengine.google.org/)
Data format	Raw and analyzed.
Data source location	Institution: University of Arkansas Agricultural Experiment Station, Arkansas Forest Resources Center, University of Arkansas at Monticello, AR, USA City/Town/Region: Monticello, Arkansas, USA State: Arkansas Country: United States Latitude and longitude (and GPS coordinates, if possible) for collected samples/data: Geospatial information is in the dataset.
Data accessibility	Repository name: Harvard Dataverse Data identification number: doi:10.7910/DVN/SWSYXG Direct URL to data: https://doi.org/10.7910/DVN/SWSYXG
Related research article	Co-submission: Zurqani, H.A. 2024. High-Resolution Forest Canopy Cover Estimation in Ecodiverse Landscape Using Machine Learning and Google Earth Engine: Validity and Reliability Assessment. Remote Sens. Appl.: Soc. Environ. 33 (2024) 101,095. https://www.sciencedirect.com/science/article/pii/S2352938523001775

## 1 Value of the Data

• The dataset provides a high-resolution (one-meter) forest canopy cover (FCC) data for the state of Arkansas, which can be used for various ecological and environmental applications.
• **Biodiversity conservation**: The high-resolution FCC data can be used to identify areas of high biodiversity, which can help prioritize conservation efforts and protect threatened and endangered species.
• **Improved forest management**: The dataset can be used to monitor changes in FCC time, identify areas of deforestation or forest degradation, and help in the planning and implementation of sustainable forest management practices.
• **Carbon sequestration**: This dataset provides valuable information for evaluating and estimating carbon sequestration rates and identifying areas with high potential for carbon storage.
• **Wildfire risk assessment**: The dataset can also be used to assess wildfire risk by identifying areas with high fuel loads and potential fire spread.
• **Urban planning**: The high-resolution FCC data can be used to assess the urban heat island effect, improve urban planning, and identify areas for green infrastructure development.
• The methodology followed in this study can be applied to any other areas around the world.

## 2 Objective

Forests have dominated most of the vegetation cover in the southern United States. However, forest environments and their inhabitants in this region have already undergone a period of remarkable rapid changes in recent decades. Thus, rapid assessment of forest disturbance is an essential source of information on local forest management planning. The advances in remote sensing and geospatial technology have significantly increased the ability to visualize and record this disturbance. This study utilized high-resolution aerial imagery and a machine learning algorithm processed and analyzed using the Google Earth Engine cloud computing platform to produce the forest canopy cover (FCC) dataset. The primary objectives of this study are to: (1) create a large-scale forest canopy cover dataset with a 1-meter spatial resolution, (2) assess the regional spatial distribution of FCC at a regional scale, and (3) investigate differences in FCC areas among the Global Forest Change [1] and U.S. Forest Service Tree Canopy Cover products at various spatial scales in Arkansas (i.e., county and city levels).

## 3 Data Description

This article presents a large-scale forest canopy cover (FCC) dataset with a 1-m spatial resolution for the state of Arkansas, USA. This dataset provides valuable information for monitoring, forecasting, and managing forest resources in Arkansas and beyond. Forest canopy cover refers to the proportion of the forest floor that is covered by the tree crowns, including the land surface directly beneath the crowns of all trees and tall shrubs in forests and urban areas (Zurqani, 2024 [2]). This parameter is critical in evaluating forest structure, biodiversity, and overall health and monitoring land use, deforestation, and reforestation changes ([3]; Liu et al., [4]; [5]). This study utilized high-resolution National Agriculture Imagery Program (NAIP) aerial imagery, which includes red, green, blue, and near-infrared bands. While optical remotely sensed data can face saturation issues in densely vegetated areas, using NAIP imagery at a 0.6-m resolution helps mitigate this problem by providing detailed information on forest canopy cover (FCC). The advantage of high spatial resolution is that it allows for a more accurate representation of individual tree crowns, reducing the likelihood of saturation in densely vegetated areas [6]. In addition, several vegetation indices were employed to address this issue, including the Normalized Difference Vegetation Index (NDVI), Enhanced Vegetation Index (EVI), Green Ratio Vegetation Index (GRVI), and Modified Soil Adjusted Vegetation Index (MSAVI). These indices provide alternative measures of vegetation health, reducing the impact of saturation in individual bands [[7], [8], [9]]. Moreover, the use of a machine learning algorithm (i.e., Random Forest classifier) enhances the classification accuracy by considering multiple spectral features and their interactions. Random Forest has been shown to handle saturation issues better than traditional classification methods, as it operates on subsets of features at each node, reducing the impact of individual saturated bands [10,11].

The proposed method offers several advantages over previous studies in FCC data generation. First, it leverages high-resolution (0.6-m) National Agriculture Imagery Program (NAIP) aerial imagery, providing a finer spatial resolution compared to coarser-resolution datasets used in some previous studies (e.g., [1]). This high-resolution imagery is essential for capturing small-scale variations in forest cover, such as individual trees within urban areas [11]. Furthermore, it emphasizes cost-effectiveness by utilizing readily available NAIP aerial imagery, making it accessible to individuals or organizations with limited resources. Second, the Random Forest (RF) classifier employed in this study contributes to the robustness, stability, and accuracy of the classification results [10]. In addition, the use of Google Earth Engine (GEE) as a cloud computing platform enhances efficiency and accessibility, allowing for rapid data processing and analysis [6]. Third, it allows for the extraction of accurate and high-resolution (one-meter) data on FCC that can be used for various purposes, such as monitoring habitats, mapping land cover, identifying areas of deforestation and reforestation, developing targeted conservation strategies, and implementing effective management plans. Additionally, it fosters an accurate evaluation of carbon sequestration potential, contributing to efforts to mitigate climate change. Last but not least, the presented approach results in accurate forest cover classification and can be easily repeated as new remote sensing layers are integrated into the GEE platform, which enables the identification of various disturbances on the earth's surface over time.

**Fig. 1:**
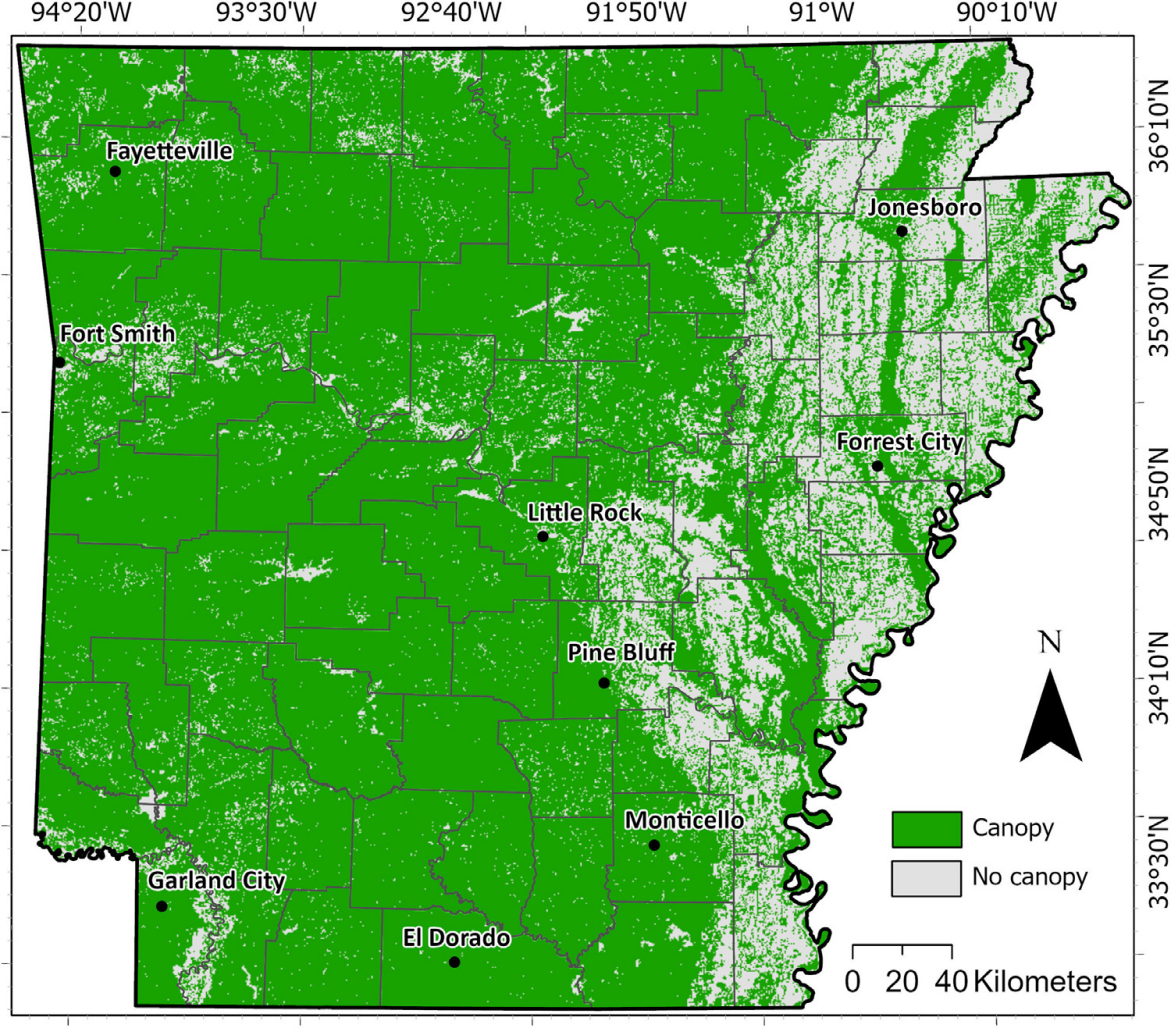
Visualization of the forest canopy cover (FCC) in Arkansas, USA extracted from NAIP imagery. Pixels are 1 m^2^.

Fig. 1. shows the distribution of FCC areas per county in Arkansas state, USA, extracted from NAIP imagery (i.e., pixels are 1 m^2^). This map reveals the detailed FCC features, such as trees within the urban areas. This high-resolution classification map is essential for understanding the spatial patterns of forest cover in the area and has important implications for the management and conservation of Arkansas’ forests.

**Fig. 2:**
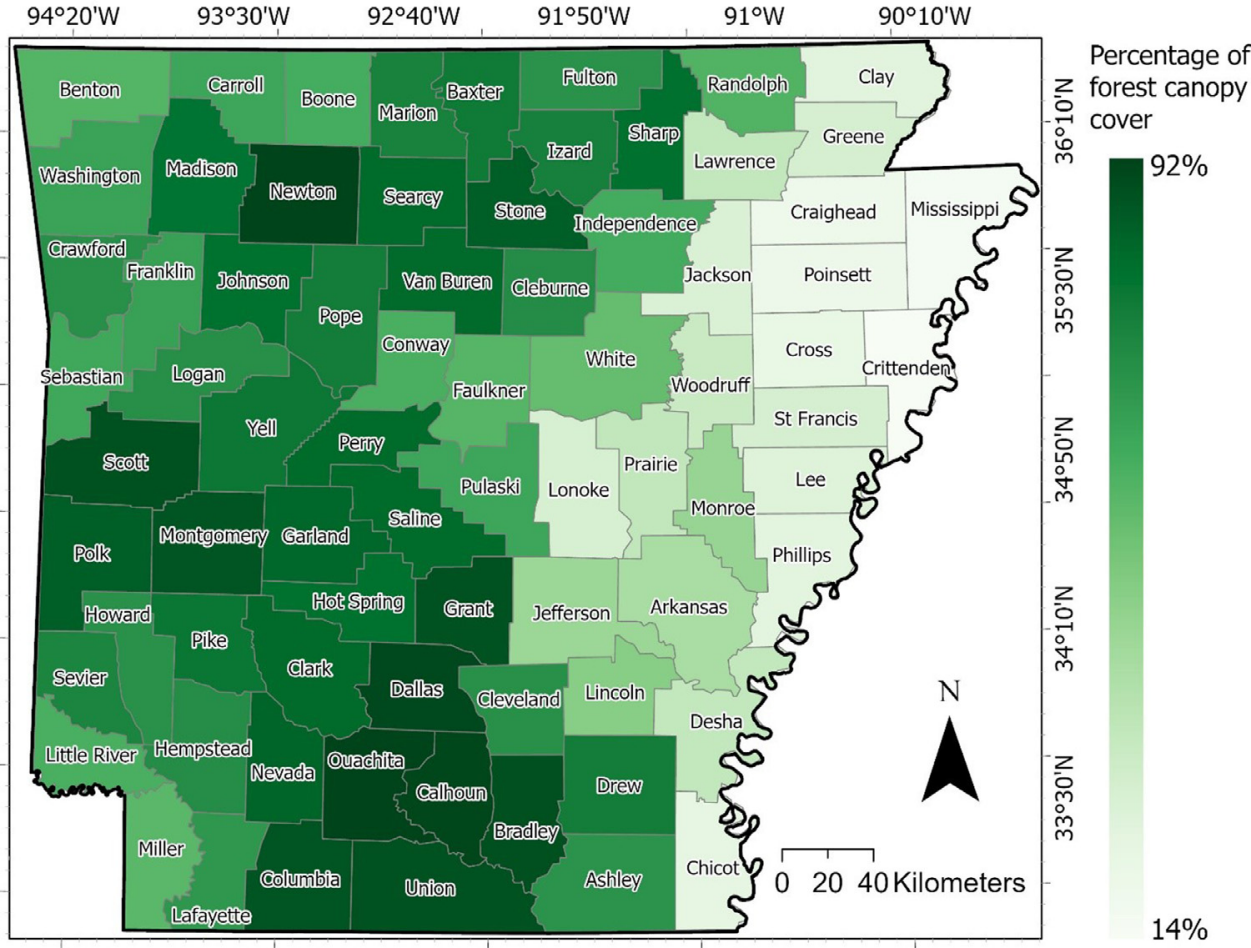
The percentage of forest canopy cover (FCC) for each county in Arkansas, USA, extracted from NAIP imagery. Pixels are 1 m^2^.

The distribution of forest canopy cover (FCC) areas per county in Arkansas state is summarized in (Supplementary Table 1). According to the classification results, the total area of the FCC in Arkansas was 86,837.10 km^2^ in 2016, which accounts for 64% of the total land area of the entire state. The FCC percentages in each county range from a low of 13.92% in Crittenden County to a high of 92.37% in Ouachita County (Fig. 2).

These results suggest that there is significant variation in FCC across different counties in this region. It is important to note that the percentage of FCC is just one aspect of a county's ecosystem and does not necessarily reflect the overall health or biodiversity of an area.

**Fig. 3:**
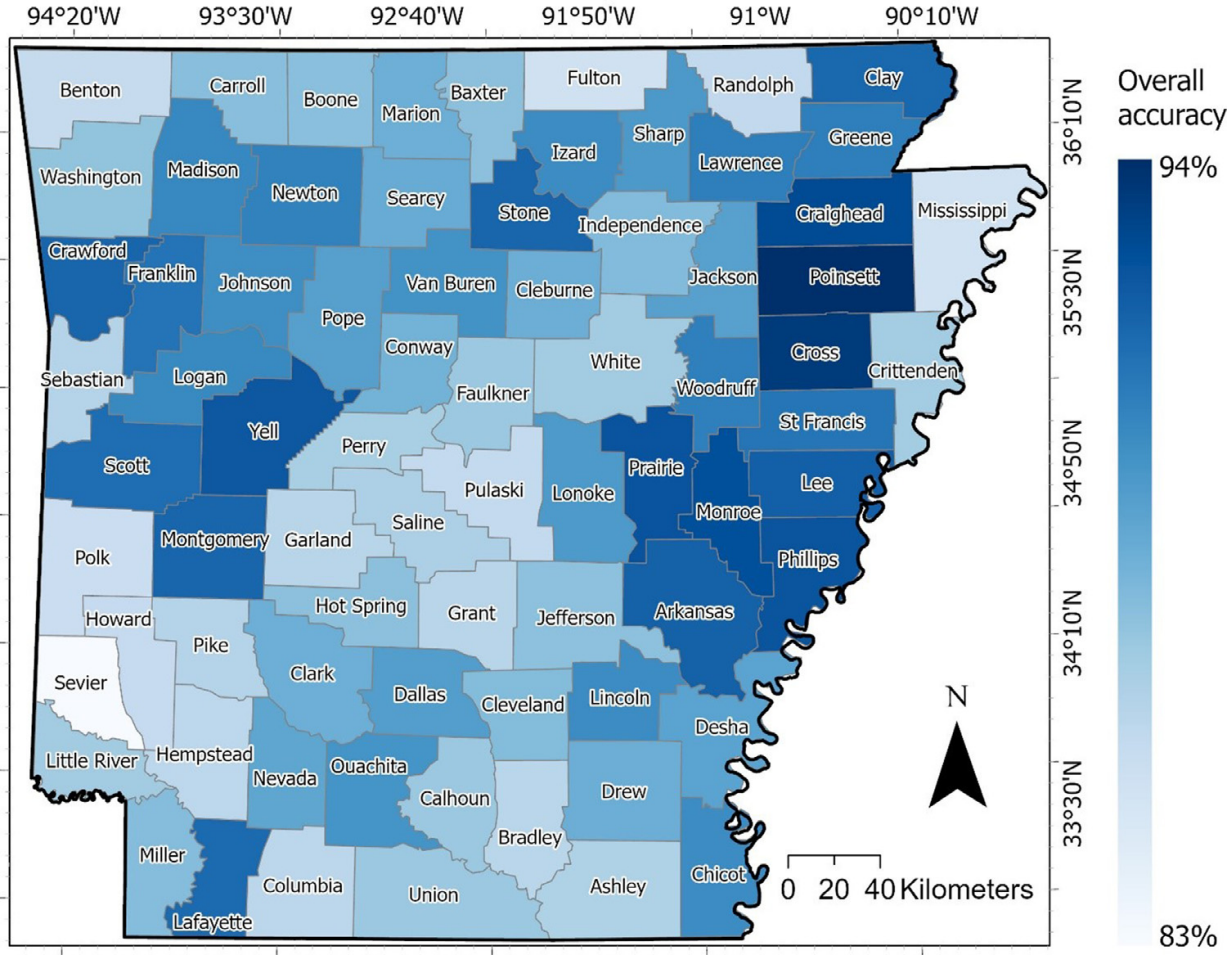
The overall accuracy of the final forest canopy cover (FCC) map for each county in Arkansas, USA.

Classification accuracies obtained from the Random Forest (RF) classifier are listed in (Supplementary Table 2). The overall accuracy for the final forest canopy cover (FCC) map ranging between 83.31% and 94.35% per county, as shown in Fig. 3. The user’s accuracy for forest and non-forest classifications within each county was between 79.09% and 95.97% and 72.98% and 95.98%, respectively. The producer's accuracy ranging from 56.95% to 97.35% for the forest and from 60.77% to 98.55% for the non-forest. Furthermore, the F1 scores for the forest and non-forest classification of all counties were also promising, with all scores being above 0.68 (Supplementary Table 3), and the Kappa coefficient ranging between 0.61and 0.85.

**Fig. 4:**
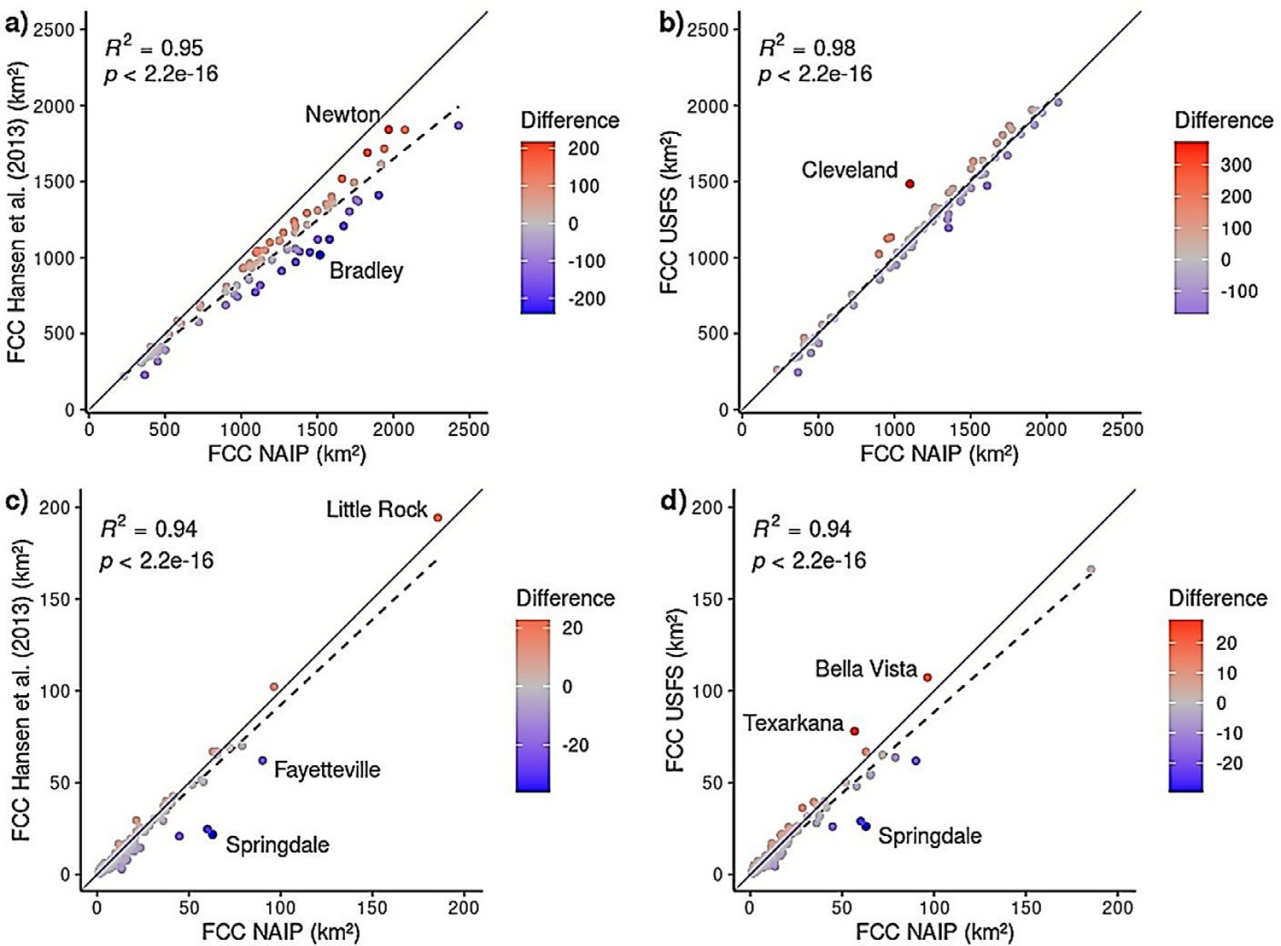
Scatter plots of the difference between the forest canopy cover (FCC) derived from NAIP imagery and the FCC from [1] and USFS products. Results are shown FCC in Arkansas: (a and b) at the county-level (*n* = 75), and (c and d) at the city-level (*n* = 500). The legend of the residual scale indicates the degree of the discrepancy, with redder colors representing more FCC areas from [1] and USFS products and bluer colors representing more FCC areas from NAIP imagery. Areas with no differences between the products will appear in gray. The dashed black line is the fitted line, and the blue 1:1 line is included as a reference.

Fig. 4a–d compares the forest canopy cover (FCC) classification results derived from NAIP imagery with the Global Forest Change [1] and U.S. Forest Service Tree Canopy Cover products at the county and the city level. If the two estimates of the FCC areas were in complete agreement, each point would fall on the diagonal line. The results indicate a strong positive correlation between this study and both the [1] and USFS products, with the R^2^ values being 0.95 and 0.98 at the county level and 0.94 at the city level, respectively (Fig. 4a–d). Only three counties—Newton and Bradley for the [1] dataset and Cleveland for the USFS dataset—showed a difference of more than 200 km^2^ between the FCC results derived from NAIP imagery and the FCC results from [1] and USFS products at the county level. Meanwhile, at the city level, six cities—Little Rock, Fayetteville, and Springdale for the [1] dataset, and Bella Vista, Texarkana, and Springdale for the USFS dataset—showed a difference of more than 20 km^2^.

The differences in forest canopy cover (FCC) estimates obtained from NAIP aerial imagery versus those obtained from [1] and USFS products are expected. The cause of these discrepancies can be attributed to various factors. For instance, NAIP imagery might not accurately represent areas with recent deforestation. In contrast, [1] and USFS products might fail to reflect changes that have occurred on the ground between the time the imagery was captured and the time the FCC results were computed. Moreover, the FCC estimates obtained from [1] and USFS products have a lower spatial resolution (30 m) compared to those derived from NAIP imagery (1 m), which could contribute to the observed disparities. Despite these discrepancies, this study demonstrates that the FCC estimates derived from NAIP imagery are highly comparable to those obtained from [1] and USFS products at the city and county levels.

## 4 Experimental Design, Materials and Methods

### 4.1 Image pre-preprocessing and classification

**Fig. 5:**
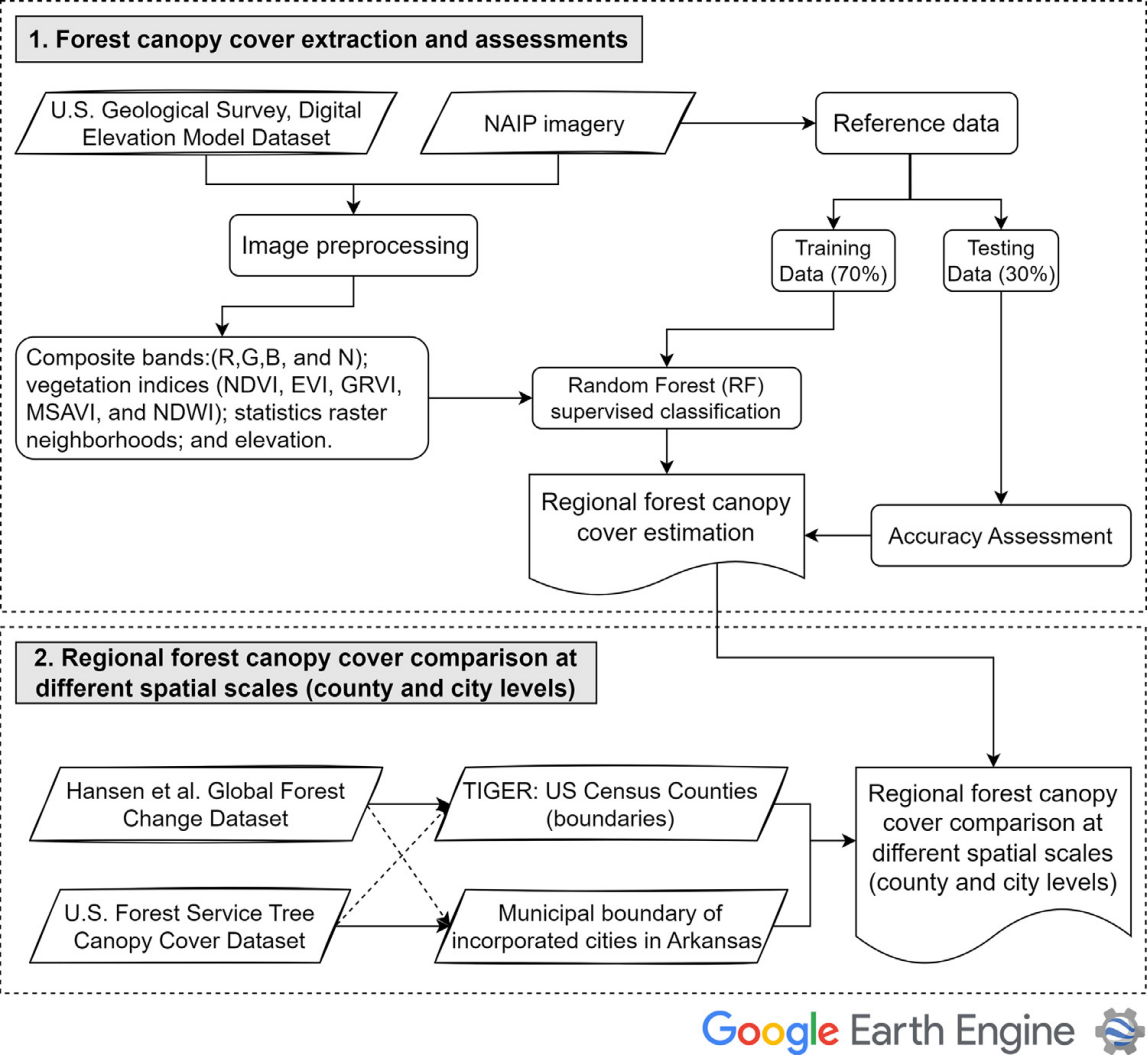
A flow diagram for data processing and the analysis steps.

Google Earth Engine leverages Google's cloud computing infrastructure platform (https://earthengine.google.org/) to dramatically reduce analysis time. It provides quick access to remote sensing products through its cloud platform, as well as pre-processing of archived data from the US Geological Survey (USGS) collection [12]. An ImageCollection of the National Agriculture Imagery Program (NAIP) was loaded and mosaicked into a single image for the dates of 2015 and 2016 (as shown in Table 1 and Fig. 5) to cover the entire state as the edges of Arkansas state were acquired in the year 2016. The composite of the NAIP imagery included the red, green, blue (RGB, 'visible spectrum'), near-infrared (NIR) bands, and the statistics image neighborhoods of these bands including the windowed entropy and texture metrics from the Gray Level Co-occurrence Matrix around each pixel [13]. The window size and shape were specified by “ee.kernel” of the 2 × 2 m neighborhood around the input pixel.

**Table 1:** Data sources and descriptions.

Data layer	Source	Spatial resolution	Date
TIGER: US Census Counties (boundaries)	Google earth engine (GEE) data provided by United States Census Bureau	–	2018
Municipal boundary of incorporated cities in Arkansas	The Arkansas GIS Office	–	2022
National Agriculture Imagery Program (NAIP)	Google earth engine (GEE) data provided by U.S. Department of Agriculture (USDA)	0.6 m	2015, 2016
U.S. Geological Survey, 3D Elevation Program 1-Meter Resolution Digital Elevation Model.	Google earth engine (GEE) data provided by U.S. Geological Survey (USGS)	1 m	2020
Hansen Global Forest Change v1.9 (2000–2021)	Google earth engine (GEE) data provided by U.S. Geological Survey (USGS)	30 m	2015
USFS Tree Canopy Cover Datasets - USDA	The U.S. Forest Service (USFS) Geospatial Technology and Applications Center (GTAC)	30 m	2016

The composite then was used to generate several indices derived from spectral band combinations that better represent vegetation greenness, such as the Normalized Difference Vegetation Index (NDVI) [14], the Enhanced Vegetation Index (EVI) [15], the Green Ratio Vegetation Index (GRVI) [16], and the Modified Soil Adjusted Vegetation Index (MSAVI) [8]. Additionally, the Normalized Difference Water Index (NDWI) [17] was used to differentiate between water and vegetated areas. All these indices were calculated for each image and stacked for later classification (Fig. 5) with the equations (Table 2). The topographic data USGS digital elevation model (i.e., elevation) was also used to better distinguish the vegetation cover and other types of land cover classes in high-terrain areas [[6], [18]].

**Table 2:** Input variables used for forest vs. non-forest classification in this study.

Input variable	Formula/Description	Data source
NAIP Imagery bands (RGB, 'visible spectrum'), and near-infrared (NIR)	–	NAIP Imagery
Normalized difference vegetation index (NDVI)	(NIR - Red) / (NIR + Red)	NAIP Imagery
Enhanced vegetation index (EVI)	2.5 * (NIR - Red) / (NIR + 6 * Red - 7.5 * Blue + 1)	NAIP Imagery
Green index (G.I.)	(NIR / Green)	NAIP Imagery
MSAVI	(2 * NIR + 1 – sqrt ((2 * NIR + 1) 2 – 8 * (NIR - R))) / 2	NAIP Imagery
Normalized difference water index (NDWI)	(Green - NIR) / (Green + NIR)	NAIP Imagery
Texture metrics from the Gray Level Co-occurrence Matrix around each pixel (glcmTexture).	*Contrast*: measures the local contrast of an image. *Savg*: Sum Average. *Ent*: Entropy. Measures the randomness of a gray-level distribution. *Dvar*: Difference variance. *Dent*: Difference entropy. *Diss*: Dissimilarity. *Inertia*: Inertia.	NAIP Imagery
Terrain elevation	–	USGS NED

The GEE platform provides a selection of classification machine learning (ML) algorithms for supervised classification, such as Random Forests (RF), Classification and Regression Trees (CART), Support Vector Machine (SVM), and Naïve Bayes [12]. For this study, a Random Forests (RF) classifier algorithm was utilized due to its robustness, stability and accuracy, which are all essential for machine learning applications. The performance of the RF classifier was further enhanced by selecting optimized parameters after visual and experimental evaluation. RF relies on two main input parameters: the number of trees and the variables at each split. It is important to note that while having a large number of trees can be beneficial, it can also be redundant if the number exceeds a certain limit. Thus, once a certain point is reached, further trees become unnecessary and do not influence the accuracy of classification (Zurqani, 2024 [2]). In this study, the input parameters for RF were adjusted as follows: {*numberOfTrees*:200, *variablesPerSplit*: 5, *minLeafPopulation*: 5, *bagFraction*: 0.5, *maxNodes*: 40, *seed*:1}.

### 4.2 Training and testing the classifier (generating reference dataset)

The reference dataset plays an essential role in the classification and validation process of remotely sensed data [19]. To produce a highly reliable reference dataset, a proportionate stratified random sample approach such as that implemented by [20] is needed to prepare and train the machine learning classification model ([21,22]; Zurqani et al., [2]). Stratified random sampling is an excellent method of choosing members of a sample when there are clearly defined subgroups in the population [23]. In this approach, the first step is to find the total area of the region of interest (i.e., the collection of pixels in the map) and then the area of each stratum (i.e., forest and non-forest). The proportion of each stratum is obtained by dividing its area by the total area of the region of interest. Finally, the sample size for each stratum is determined by multiplying the proportion by the desired sample size, as shown in the following equation.

(1) $$Stratum\;sample\;size=\frac{Area\;of\;stratum\;}{Total\;area\;of\;the\;region\;of\;interest}*\left(Desired\;sample\;size\right)$$

In this study, *stratifiedSample* method and visual interpretation technique were used to produce more than 2000 reference points for each county in Arkansas using the Global Forest Change [1] dataset and the 0.6-m spatial resolution National Agriculture Imagery Program (NAIP) aerial imagery with a minimum of no less than 450 reference points for each of the two categories (i.e., forest and non-forest). This reference dataset was then split randomly into a training dataset set consisting of 70% of the observations and a testing dataset with the remaining 30% of the observations. The training dataset was used to train the supervised classifier algorithm, and the testing data set was used to measure the accuracy of the results.

### 4.3 Accuracy assessment

Accuracy assessments have been identified as an important part of evaluating the reliability of remotely sensed data for particular applications [[24], [25], [26]]. The accuracy assessment technique enables a comparison between single-pixel values in a raster layer and the reference pixels for which the class is defined [6]. The testing dataset, which represents one-third of the observations (i.e., reference locations) as shown in (Fig. 2), was utilized to validate the produced forest canopy cover (FCC) map. Following previous studies [27,28], a confusion matrix was calculated to evaluate the accuracy of the results, looking at the producer’s accuracy, user’s accuracy, the overall accuracy, F1 score [6], and kappa coefficient [24], as shown in equations. (2), (3), (4), (5), (6), respectively. The kappa coefficient reflects the difference between actual agreement and agreement expected by chance, as shown in equation. (5), and F1 scores measure how good the classifier is in the context of both producer's and user's, weighting the average of producer's and user's as shown in equation. (6):

(2) $$Producer^{\prime }s\;accuracy=\frac{Total\;number\;of\;classified\;points\;that\;agree\;with\;reference\;data}{Total\;number\;of\;reference\;points\;for\;that\;class}$$

(3) $$User^{\prime }s\;accuracy=\frac{Total\;number\;of\;classified\;points\;that\;agree\;with\;referencedata}{Total\;number\;of\;classified\;points\;for\;that\;class}$$

(4) $$Overall\;accuracy=\frac{Total\;correctly\;classified\;pixels}{Total\;number\;of\;reference\;pixels\;in\;the\;error\;matrix\left(N\right)}$$

(5) $$Kappa\;coefficient=\frac{observed\;accuracy-agreement\;chane}{1-agreement\;chane}$$

(6) $$F1\;score\;=\;2\cdot \frac{user^{\prime }s\;\cdot \;producer^{\prime }s}{user^{\prime }s\;+\;producer^{\prime }s}$$

## Ethics Statement

The authors declare that the work did not involve the use of human subjects nor animal experiments.

## CRediT Author Statement

**Hamdi Zurqani**: Conceptualization, Methodology, Software, Data curation, Writing- Original draft preparation, Visualization, Investigation, Writing- Reviewing and Editing. The author has read and agreed to the published version of the manuscript.

## Data Availability

One meter Forest Canopy Cover (FCC) for Arkansas, USA (Reference data) (Dataverse)

## Appendix Supplementary materials

Image, application 1

Image, application 2
