# Supplementary material for: The first generation of a regional-scale 1-m forest canopy cover dataset using machine learning and google earth engine cloud computing platform: A case study of Arkansas, USA
Source: Data Brief. 2023 Dec 30;52:109986. doi: 10.1016/j.dib.2023.109986 (PMC10827392; doi:10.1016/j.dib.2023.109986)
Supplement: Supplementary file 1 [file mmc1.docx]

**Supplementary Table 1**. The distribution of forest canopy cover areas per county in Arkansas state, USA.

| **County name** | **Forest (Km^2^)** | **Non-forest**  **(Km^2^)** | **Total (Km^2^)** | **Forest (%)** | **Non-forest (%)** | **Total (%)** |
| --- | --- | --- | --- | --- | --- | --- |
| Phillips | 452.9165 | 1430.78 | 1883.696 | 24.04403 | 75.95597 | 100 |
| Calhoun | 1502.736 | 135.1565 | 1637.893 | 91.74815 | 8.251852 | 100 |
| Union | 2427.348 | 305.594 | 2732.942 | 88.81813 | 11.18187 | 100 |
| Columbia | 1754.488 | 229.6242 | 1984.112 | 88.42685 | 11.57315 | 100 |
| Ouachita | 1769.916 | 146.0289 | 1915.945 | 92.37823 | 7.621769 | 100 |
| Clark | 1903.257 | 383.2558 | 2286.513 | 83.23842 | 16.76158 | 100 |
| Nevada | 1357.679 | 249.7779 | 1607.456 | 84.4613 | 15.5387 | 100 |
| Hempstead | 1384.059 | 535.572 | 1919.631 | 72.10027 | 27.89973 | 100 |
| Miller | 954.7645 | 696.6005 | 1651.365 | 57.81669 | 42.18331 | 100 |
| Little River | 897.7665 | 564.754 | 1462.52 | 61.38488 | 38.61512 | 100 |
| Newton | 1968.733 | 163.3349 | 2132.068 | 92.33913 | 7.660867 | 100 |
| Pope | 1661.818 | 489.8705 | 2151.688 | 77.2332 | 22.7668 | 100 |
| Yell | 1939.625 | 517.9241 | 2457.549 | 78.92518 | 21.07482 | 100 |
| Baxter | 1188.071 | 331.4284 | 1519.5 | 78.18832 | 21.81168 | 100 |
| Boone | 970.3142 | 588.8115 | 1559.126 | 62.23451 | 37.76549 | 100 |
| Independence | 1252.323 | 745.8805 | 1998.203 | 62.67243 | 37.32757 | 100 |
| Mississippi | 366.1614 | 2015.957 | 2382.118 | 15.37126 | 84.62874 | 100 |
| Washington | 1608.702 | 857.9524 | 2466.655 | 65.21798 | 34.78202 | 100 |
| Benton | 1355.228 | 933.9561 | 2289.184 | 59.20135 | 40.79865 | 100 |
| Madison | 1741.464 | 426.5368 | 2168.001 | 80.3258 | 19.6742 | 100 |
| Crawford | 1113.659 | 448.5676 | 1562.227 | 71.28666 | 28.71334 | 100 |
| Sebastian | 902.8733 | 514.3498 | 1417.223 | 63.70721 | 36.29279 | 100 |
| Hot Spring | 1303.121 | 308.3063 | 1611.428 | 80.86751 | 19.13249 | 100 |
| Garland | 1593.655 | 308.9907 | 1902.645 | 83.75994 | 16.24006 | 100 |
| Craighead | 344.6035 | 1501.352 | 1845.956 | 18.66802 | 81.33198 | 100 |
| Poinsett | 371.5093 | 1606.152 | 1977.662 | 18.78528 | 81.21472 | 100 |
| Greene | 432.8998 | 1068.07 | 1500.97 | 28.84134 | 71.15866 | 100 |
| Lincoln | 721.2723 | 760.6894 | 1481.962 | 48.6701 | 51.3299 | 100 |
| Jefferson | 1051.041 | 1315.632 | 2366.672 | 44.41006 | 55.58994 | 100 |
| Lonoke | 581.8316 | 1495.636 | 2077.467 | 28.00678 | 71.99322 | 100 |
| Cleveland | 1101.466 | 449.2636 | 1550.729 | 71.02888 | 28.97112 | 100 |
| Pulaski | 1348.29 | 743.5343 | 2091.825 | 64.45523 | 35.54477 | 100 |
| Grant | 1451.363 | 187.905 | 1639.268 | 88.53726 | 11.46274 | 100 |
| Saline | 1569.689 | 322.278 | 1891.967 | 82.96598 | 17.03402 | 100 |
| Perry | 1201.744 | 250.0138 | 1451.758 | 82.77855 | 17.22145 | 100 |
| Faulkner | 1010.336 | 709.5967 | 1719.932 | 58.74276 | 41.25724 | 100 |
| White | 1502.112 | 1197.039 | 2699.151 | 55.65128 | 44.34872 | 100 |
| St Francis | 477.6642 | 1186.507 | 1664.172 | 28.70282 | 71.29718 | 100 |
| Crittenden | 229.5148 | 1418.53 | 1648.045 | 13.92649 | 86.07351 | 100 |
| Desha | 729.2491 | 1393.123 | 2122.372 | 34.36009 | 65.63991 | 100 |
| Arkansas | 1097.475 | 1579.922 | 2677.397 | 40.99036 | 59.00964 | 100 |
| Monroe | 730.8455 | 878.319 | 1609.164 | 45.4177 | 54.5823 | 100 |
| Drew | 1672.833 | 491.5199 | 2164.352 | 77.29021 | 22.70979 | 100 |
| Ashley | 1710.765 | 725.1872 | 2435.953 | 70.22983 | 29.77017 | 100 |
| Prairie | 603.5523 | 1146.266 | 1749.818 | 34.49229 | 65.50771 | 100 |
| Dallas | 1579.959 | 150.428 | 1730.387 | 91.30668 | 8.693316 | 100 |
| Bradley | 1517.408 | 173.6355 | 1691.043 | 89.73205 | 10.26795 | 100 |
| Lafayette | 976.104 | 435.6557 | 1411.76 | 69.14095 | 30.85905 | 100 |
| Pike | 1265.65 | 324.9442 | 1590.594 | 79.57089 | 20.42911 | 100 |
| Montgomery | 1829.39 | 243.3181 | 2072.708 | 88.26086 | 11.73914 | 100 |
| Howard | 1093.141 | 448.7119 | 1541.853 | 70.89789 | 29.10211 | 100 |
| Sevier | 1124.686 | 380.7326 | 1505.419 | 74.70919 | 25.29081 | 100 |
| Polk | 1918.58 | 315.0858 | 2233.665 | 85.89378 | 14.10622 | 100 |
| Logan | 1354.012 | 540.7013 | 1894.713 | 71.46263 | 28.53737 | 100 |
| Franklin | 1056.107 | 548.7409 | 1604.848 | 65.8073 | 34.1927 | 100 |
| Johnson | 1432.279 | 336.0237 | 1768.303 | 80.99739 | 19.00261 | 100 |
| Conway | 898.9969 | 568.6612 | 1467.658 | 61.25384 | 38.74616 | 100 |
| Van Buren | 1559.326 | 316.5495 | 1875.876 | 83.12524 | 16.87476 | 100 |
| Searcy | 1434.359 | 297.083 | 1731.442 | 82.84187 | 17.15813 | 100 |
| Marion | 1249.337 | 408.9927 | 1658.33 | 75.33707 | 24.66293 | 100 |
| Fulton | 1132.488 | 474.0652 | 1606.553 | 70.49178 | 29.50822 | 100 |
| Izard | 1156.493 | 356.1015 | 1512.595 | 76.45758 | 23.54242 | 100 |
| Sharp | 1276.804 | 293.8586 | 1570.662 | 81.29079 | 18.70921 | 100 |
| Randolph | 1017.331 | 680.4659 | 1697.797 | 59.92065 | 40.07935 | 100 |
| Lawrence | 525.3091 | 1008.871 | 1534.18 | 34.24038 | 65.75962 | 100 |
| Stone | 1356.313 | 222.1223 | 1578.435 | 85.92769 | 14.07231 | 100 |
| Cleburne | 1110.174 | 422.8855 | 1533.06 | 72.41559 | 27.58441 | 100 |
| Woodruff | 501.8236 | 1036.532 | 1538.355 | 32.62078 | 67.37922 | 100 |
| Jackson | 450.8152 | 1210.671 | 1661.486 | 27.13325 | 72.86675 | 100 |
| Cross | 346.0973 | 1265.674 | 1611.771 | 21.47311 | 78.52689 | 100 |
| Lee | 408.8318 | 1194.863 | 1603.695 | 25.49312 | 74.50688 | 100 |
| Clay | 406.3479 | 1254.804 | 1661.152 | 24.46181 | 75.53819 | 100 |
| Chicot | 405.9371 | 1383.018 | 1788.955 | 22.69129 | 77.30871 | 100 |
| Carroll | 1066.72 | 587.2388 | 1653.959 | 64.49496 | 35.50504 | 100 |
| Scott | 2075.544 | 250.3737 | 2325.918 | 89.23549 | 10.76451 | 100 |
