# Supplementary material for: The first generation of a regional-scale 1-m forest canopy cover dataset using machine learning and google earth engine cloud computing platform: A case study of Arkansas, USA
Source: Data Brief. 2023 Dec 30;52:109986. doi: 10.1016/j.dib.2023.109986 (PMC10827392; doi:10.1016/j.dib.2023.109986)
Supplement: Supplementary file 2 [file mmc2.docx]

**Supplementary Table 2**. Forest canopy cover extraction accuracies obtained from the Random Forest (RF) classifier.

| **County name** | **Overall accuracy** | **Kappa coefficient** | **Producer’s accuracy (non-forest)** | **Producer’s accuracy (forest)** | **User’s accuracy (non-forest)** | **User’s accuracy (forest)** | **F1 score (non-forest)** | **F1 score (forest)** |
| --- | --- | --- | --- | --- | --- | --- | --- | --- |
| Arkansas | 92.24 | 0.83 | 92.81 | 91.13 | 95.36 | 86.59 | 0.94 | 0.89 |
| Ashley | 86.81 | 0.72 | 80.56 | 90.77 | 84.67 | 88.06 | 0.83 | 0.89 |
| Baxter | 87.94 | 0.72 | 78.28 | 92.49 | 83.04 | 90.06 | 0.81 | 0.91 |
| Benton | 86.02 | 0.72 | 83.81 | 88.96 | 90.96 | 80.57 | 0.87 | 0.85 |
| Boone | 87.97 | 0.76 | 87.77 | 88.2 | 89.71 | 86.03 | 0.89 | 0.87 |
| Bradley | 86.46 | 0.62 | 69.39 | 91.86 | 72.98 | 90.45 | 0.71 | 0.91 |
| Calhoun | 87.52 | 0.63 | 65.27 | 94.52 | 78.99 | 89.62 | 0.71 | 0.92 |
| Carroll | 88.02 | 0.76 | 86.51 | 89.3 | 87.28 | 88.64 | 0.87 | 0.89 |
| Chicot | 90.41 | 0.65 | 96.2 | 64.44 | 92.38 | 79.09 | 0.94 | 0.71 |
| Clark | 88.76 | 0.72 | 76.33 | 93.86 | 83.6 | 90.63 | 0.8 | 0.92 |
| Clay | 91.97 | 0.74 | 97.5 | 71.18 | 92.71 | 88.32 | 0.95 | 0.79 |
| Cleburne | 88.81 | 0.76 | 84.14 | 91.76 | 86.52 | 90.19 | 0.85 | 0.91 |
| Cleveland | 88.14 | 0.65 | 67.88 | 94.38 | 78.81 | 90.51 | 0.73 | 0.92 |
| Columbia | 86.43 | 0.64 | 66.28 | 94.18 | 81.43 | 87.89 | 0.73 | 0.91 |
| Conway | 88.58 | 0.77 | 89.52 | 87.53 | 88.89 | 88.24 | 0.89 | 0.88 |
| Craighead | 93.22 | 0.71 | 97.5 | 68.47 | 94.7 | 82.61 | 0.96 | 0.75 |
| Crawford | 92.07 | 0.83 | 93.38 | 91.31 | 86.17 | 95.97 | 0.9 | 0.94 |
| Crittenden | 87.23 | 0.61 | 97 | 56.95 | 87.47 | 86 | 0.92 | 0.68 |
| Cross | 93.86 | 0.76 | 98.55 | 70.87 | 94.31 | 90.91 | 0.96 | 0.8 |
| Dallas | 89.64 | 0.66 | 64.24 | 96.34 | 82.2 | 91.09 | 0.72 | 0.94 |
| Desha | 89.39 | 0.75 | 93.58 | 80.25 | 91.18 | 85.15 | 0.92 | 0.83 |
| Drew | 88.8 | 0.75 | 78.24 | 94.47 | 88.36 | 89 | 0.83 | 0.92 |
| Faulkner | 87.5 | 0.74 | 90.74 | 82.72 | 88.55 | 85.86 | 0.9 | 0.84 |
| Franklin | 91.45 | 0.83 | 89.14 | 93.41 | 92.04 | 90.97 | 0.91 | 0.92 |
| Fulton | 85.5 | 0.7 | 78.04 | 90.74 | 85.56 | 85.46 | 0.82 | 0.88 |
| Garland | 86.55 | 0.67 | 67.87 | 95.55 | 88.02 | 86.06 | 0.77 | 0.91 |
| Grant | 86.56 | 0.62 | 68.65 | 92.11 | 72.99 | 90.44 | 0.71 | 0.91 |
| Greene | 91.01 | 0.74 | 96.73 | 72.67 | 91.9 | 87.41 | 0.94 | 0.79 |
| Hempstead | 86.38 | 0.72 | 81.25 | 90.27 | 86.39 | 86.36 | 0.84 | 0.88 |
| Hot Spring | 87.95 | 0.71 | 75.1 | 93.79 | 84.58 | 89.25 | 0.8 | 0.92 |
| Howard | 86.08 | 0.72 | 83.76 | 88.13 | 86.22 | 85.96 | 0.85 | 0.87 |
| Independence | 88.19 | 0.76 | 87.89 | 88.47 | 87.89 | 88.47 | 0.88 | 0.89 |
| Izard | 90.42 | 0.79 | 87.19 | 92.05 | 84.74 | 93.42 | 0.86 | 0.93 |
| Jackson | 89.45 | 0.74 | 93.99 | 78.49 | 91.34 | 84.39 | 0.93 | 0.81 |
| Jefferson | 87.91 | 0.73 | 93.68 | 77.14 | 88.43 | 86.75 | 0.91 | 0.82 |
| Johnson | 90.32 | 0.78 | 78.29 | 96.57 | 92.24 | 89.53 | 0.85 | 0.93 |
| Lafayette | 91.86 | 0.83 | 86.69 | 95.37 | 92.71 | 91.35 | 0.9 | 0.93 |
| Lawrence | 91.02 | 0.77 | 95.4 | 79.21 | 92.51 | 86.49 | 0.94 | 0.83 |
| Lee | 92.33 | 0.76 | 98.03 | 72.25 | 92.56 | 91.24 | 0.95 | 0.81 |
| Lincoln | 90.43 | 0.8 | 91.06 | 89.54 | 92.54 | 87.54 | 0.92 | 0.89 |
| Little River | 87.3 | 0.75 | 91.03 | 83.84 | 83.96 | 90.96 | 0.87 | 0.87 |
| Logan | 90.55 | 0.81 | 85.13 | 94.69 | 92.44 | 89.29 | 0.89 | 0.92 |
| Lonoke | 89.81 | 0.7 | 95.8 | 70.29 | 91.32 | 83.67 | 0.94 | 0.76 |
| Madison | 90.64 | 0.77 | 78.51 | 95.89 | 89.2 | 91.16 | 0.84 | 0.94 |
| Marion | 88.75 | 0.74 | 78.54 | 93.95 | 86.86 | 89.57 | 0.83 | 0.92 |
| Miller | 88.09 | 0.76 | 91.11 | 84.06 | 88.42 | 87.61 | 0.9 | 0.86 |
| Mississippi | 85.45 | 0.62 | 94.35 | 63.58 | 86.42 | 82.09 | 0.9 | 0.72 |
| Monroe | 93 | 0.85 | 93.71 | 91.84 | 94.9 | 90 | 0.94 | 0.91 |
| Montgomery | 92.04 | 0.74 | 71.71 | 97.18 | 86.51 | 93.15 | 0.78 | 0.95 |
| Nevada | 89.23 | 0.73 | 77.68 | 93.81 | 83.25 | 91.38 | 0.8 | 0.93 |
| Newton | 90.88 | 0.77 | 78.61 | 95.58 | 87.18 | 92.11 | 0.83 | 0.94 |
| Ouachita | 90.05 | 0.62 | 60.77 | 96.15 | 76.7 | 92.17 | 0.68 | 0.94 |
| Perry | 87.06 | 0.69 | 71.98 | 94.36 | 86.08 | 87.43 | 0.78 | 0.91 |
| Phillips | 92.79 | 0.71 | 96.59 | 71.79 | 94.98 | 79.25 | 0.96 | 0.75 |
| Pike | 86.64 | 0.7 | 75.94 | 92.83 | 85.96 | 86.97 | 0.81 | 0.9 |
| Poinsett | 94.35 | 0.75 | 97.55 | 73.96 | 95.98 | 82.56 | 0.97 | 0.78 |
| Polk | 85.77 | 0.65 | 68.12 | 93.4 | 81.68 | 87.15 | 0.74 | 0.9 |
| Pope | 89.49 | 0.76 | 85.02 | 91.56 | 82.35 | 92.95 | 0.84 | 0.92 |
| Prairie | 92.88 | 0.83 | 95.56 | 86.58 | 94.34 | 89.29 | 0.95 | 0.88 |
| Pulaski | 86.11 | 0.72 | 85.28 | 87.09 | 88.65 | 83.33 | 0.87 | 0.85 |
| Randolph | 86.21 | 0.72 | 82.7 | 89.82 | 89.29 | 83.5 | 0.86 | 0.87 |
| Saline | 86.96 | 0.71 | 79.22 | 91.06 | 82.45 | 89.21 | 0.81 | 0.9 |
| Scott | 91.69 | 0.74 | 71.08 | 97.35 | 88.06 | 92.45 | 0.79 | 0.95 |
| Searcy | 88.93 | 0.73 | 76.44 | 94.38 | 85.57 | 90.19 | 0.81 | 0.92 |
| Sebastian | 86.65 | 0.73 | 89.68 | 83.43 | 85.18 | 88.39 | 0.87 | 0.86 |
| Sevier | 83.31 | 0.66 | 78.77 | 87.17 | 83.93 | 82.84 | 0.81 | 0.85 |
| Sharp | 89.86 | 0.75 | 76.09 | 95.73 | 88.38 | 90.37 | 0.82 | 0.93 |
| St Francis | 91.41 | 0.74 | 96.59 | 73.45 | 92.67 | 86.09 | 0.95 | 0.79 |
| Stone | 92.07 | 0.77 | 78.16 | 96.41 | 87.18 | 93.39 | 0.82 | 0.95 |
| Union | 87.5 | 0.66 | 67.97 | 94.7 | 82.54 | 88.91 | 0.75 | 0.92 |
| Van Buren | 90.13 | 0.75 | 75.76 | 96.29 | 89.74 | 90.26 | 0.82 | 0.93 |
| Washington | 87.79 | 0.76 | 89.37 | 86.35 | 85.67 | 89.89 | 0.88 | 0.88 |
| White | 87.29 | 0.74 | 91.12 | 82.62 | 86.47 | 88.41 | 0.89 | 0.85 |
| Woodruff | 90.97 | 0.76 | 94.04 | 82.12 | 93.82 | 82.67 | 0.94 | 0.82 |
| Yell | 92.65 | 0.82 | 83.19 | 96.74 | 91.71 | 93 | 0.87 | 0.95 |
